# Competition and integration in health care reform

**DOI:** 10.5334/ijic.965

**Published:** 2012-06-15

**Authors:** Chris Ham

**Affiliations:** The King’s Fund, 11-13 Cavendish Square, W1G 0AN, London, UK

**Keywords:** integration, competition, health care reform, integrated care

## Abstract

There is a growing but still fragile understanding that competition and integration are not necessarily in conflict and can be used together. In one version, this might mean using competition to drive improvements in performance in planned care, and promoting integration to do so in relation to unplanned care and care for people with complex needs. In another, it entails arguing that competition between integrated systems might offer the best of all worlds, if policies can be designed to support evolution in that direction. This paper suggests that a bundle of policy interventions is needed to support the evolution of integrated systems of care. It examines how policies might be crafted to make this happen; How to avoid the wrong kind of integration to develop; and, how can policy-makers enable *competition* between integrated systems.

The pendulum law that shapes health policy swings back and forth between competition and integration in a regular but unpredictable motion. England is a case in point. The election of the Conservative/Liberal Democrat coalition government in 2010 led to a renewed interest in competition but recently this has been modified by recognition that integration also has a part to play in improving the performance of the NHS.

The twists and turns of English health policy can be explained in three ways. First, they reflect real and legitimate differences among politicians about the best way of running planned health care systems. Although traditionally politicians on the centre right favoured competition and those on the centre left argued for collaboration and integration, such simple distinctions no longer hold. Advocates of competition can be found across the spectrum as indeed can supporters of integration, the balance between the two shifting even under governments controlled by the same party.

The second explanation of the swinging pendulum is genuine uncertainty about the effectiveness of different policies in bringing about improvements in performance. Markets have often failed to deliver the results their advocates have promised but equally the evidence for integrated care is mixed. Hardly surprising therefore that politicians who become frustrated at the impact of one approach search out for alternatives, especially when they are operating to short timescales.

The third reason reflects a growing but still fragile understanding that competition and integration are not necessarily in conflict and can be used together. In one version, this might mean using competition to drive improvements in performance in planned care, and promoting integration to do so in relation to unplanned care and care for people with complex needs. In another, it entails arguing that competition between integrated systems might offer the best of all worlds, if policies can be designed to support evolution in that direction.

This argument is at the heart of the analysis put forward by Clayton Christensen and his fellow authors in their critique of medical care in the US [1]. Their contention is that outmoded business models need to be superseded by innovations in care that reward health care providers for keeping people well rather than paying them for treating sickness. Christensen and his collaborators maintain that integrated systems like Kaiser Permanente and Intermountain Health Care are more successful at doing this than the fragmented forms of care that are much more common in the US.

They go on to note that at points of fundamental change costs are driven down and value is increased not by competition itself but by disruptive competition. In their view, disruptive competition is best achieved by encouraging more systems like Kaiser Permanente to enter the market. The point about these systems is that in combining responsibility for funding and providing care they have incentives aligned to deliver services in the most appropriate settings because they are rewarded for keeping well and are in effect penalised when they fail to do so.

The interest in the US in developing accountable care organisations (ACOs) is closely related to these arguments, although it remains an open question as to whether ACOs will migrate from health policy journals to the consulting room. The limited penetration of integrated systems in the US, which cover only about 5% of the insured population, serves as a cautionary tale. If fragmented care rather than integrated care is the norm in both market based systems and often in planned systems, a herculean effort linked to smart policy design will be needed to buck the trend.

How then might policies be crafted to make this happen? Recent work by The King’s Fund and the Nuffield Trust has outlined what needs to be done in England to create a supportive policy context [2]. One of the most important changes concerns how care is paid for, involving a shift away from funding activity to paying for good outcomes. Experience elsewhere in Europe may help to inform what needs to be done, as in the use in the Netherlands of payment systems focused on diseases like diabetes and designed to encourage care to be provided in the right place at the right time [3].

Also essential is to ensure that regulation supports integrated care by focusing on how well organisations work together to achieve these outcomes. This in turn hinges on finding out whether patients and service users experience care that is well coordinated around their needs through regular surveys that assess progress in delivering integrated care. Planned systems can use the results of patient surveys to improve performance through transparent reporting of these results and active performance management.

To make these points is to argue that a bundle of policy interventions is needed to support the evolution of integrated systems of care. As this happens, there is a risk that ‘the wrong kind of integration’ may emerge unless policy-makers think two or three steps ahead. This is a clear risk in countries like Germany and the Netherlands where the interest in integrated care has a strong disease-based focus.

High performing integrated systems do pay attention to people with single diseases but they do so in the context of a concern for the population they serve in which their primary aim is to provide coordinated care for all. Kaiser Permanente’s ‘complete care’ approach illustrates this, the aim being to understand the needs of all members and to help them live healthy lives whatever their disease profile. The idea of population health management expresses this commitment and emphasises the value of a broad rather than narrow interpretation of integration.

This is important in that the biggest challenge facing all health care systems, whether based on planning or markets, is to better meet the needs of ageing populations in which people with multi morbidities are responsible for a high proportion of service use and cost. Treating people and not diseases therefore has to be the priority for the future both to contain costs and to improve outcomes. Disease-based integration risks creating new silos to replace old ones and failing to support the rapid and radical reorientation that is needed.

How then can policy-makers enable *competition* between integrated systems? Part of the answer lies in ensuring that market regulators do not see integration as a form of collusion between providers that inhibits the invisible hand working its magic. In this respect, it is heartening that chairman and chief executive of Monitor, the market regulator of the English NHS, has gone on record in offering support to integrated care where it will bring benefits [4].

Also important is designing the market in a way that supports different forms of integrated care to evolve rather than prescribing one approach at the outset. As Harford has argued, many of today’s challenges cannot be tackled through readymade solutions and a willingness to improvise and innovate is therefore essential [5]. In planned health care systems this means overcoming deep seated tendencies to plan and prescribe and also to tolerate the likelihood of failure as the price of ultimate success.

Another ingredient is a willingness to support innovative and ambitious approaches to integrated care and to do so over a period of years. Too often policy-makers promote integration through small-scale pilots that are evaluated over a short timescale. A good example is the integrated care programme set up in England in 2008, the results of which have just been released. These results may be a disappointment to the advocates of integration but they are hardly surprising given their level of ambition and the limited time allowed to the pilots involved in the programme to make a difference.

As the health policy pendulum takes a new turn, the focus of debate needs to shift away from simple and unhelpful dichotomies such as that which pitches the advocates of competition against the supporters of integration. Both have a part to play and the challenge is to craft a set of reforms to promote the right kind of competition and integration to emerge at the scale and pace needed in future.

## About the author

Chris Ham took up his post as Chief Executive of The King’s Fund in April 2010.

He has been professor of health policy and management at the University of Birmingham, England, since 1992. From 2000 to 2004 he was seconded to the Department of Health where he was director of the strategy unit, working with Ministers on NHS reform. Chris is the author of 20 books and numerous articles about health policy and management. His work focuses on the use of research evidence to inform policy and management decisions in areas such as health care reform, chronic care, primary care, integrated care, performance improvement and leadership.

Chris has advised the WHO and the World Bank and has served as a consultant to governments in a number of countries. He is an honorary fellow of the Royal College of Physicians of London and of the Royal College of General Practitioners, an honorary professor at the London School of Hygiene and Tropical Medicine, a companion of the Institute of Healthcare Management, a senior associate of the Nuffield Trust, and a visiting professor at the University of Surrey.

In 2004 he was awarded a CBE for his services to the National Health Service.
